# Student Assessments of Early Childhood Caries in Some Informal Settlements of Cape Town: A 10‐Year Series of Cross‐Sectional Studies

**DOI:** 10.1002/puh2.70049

**Published:** 2025-04-17

**Authors:** Larisa Krekmanova, Neil Myburgh, Ted Lundgren

**Affiliations:** ^1^ Department of Pediatric Dentistry Institute of Odontology at the Sahlgrenska Academy University of Gothenburg Gothenburg Sweden; ^2^ Department of Community Dentistry University of the Western Cape Cape Town South Africa

**Keywords:** children | dmft | Early Childhood Caries | primary dentition | pufa | Significant Caries Index (SiC)

## Abstract

**Aim:**

To assess Early Childhood Caries by measuring the dmft, Significant Caries Index (SiC), pufa, and caries‐free status in informal settlements outside Cape Town, South Africa over a 10‐year period.

**Method:**

A series of cross‐sectional studies over a 10‐year period (2009–2019). Surveys were carried out in preschools in informal settlements. A total of 5090 children aged from 1 to 5 years of age were examined.

**Results:**

For 1‐ to 3‐year‐olds, the mean dmft was 4.33 ± 1.55, and for 4‐ and 5‐year‐olds, it was 6.34 ± 1.48. The difference in dmft for 1‐ to 3‐year‐olds increased but did not differ statistically between 2009 and 2019. For 4–5 years old, there was an increase over time. For 1‐ to 3‐year‐olds, the mean pufa was 0.34 ± 0.28, and for 4‐ to 5‐year‐olds, it was 0.63 ± 0.59. For 1‐ to 3‐ and for 4‐ to 5‐year‐olds, there was a statistically significant, lower pufa 2009–2019. For 1‐ to 3‐year‐olds, the mean SiC was 11.0 ± 2.35, and for 4‐ to 5‐year‐olds, it was 12.05 ± 1.89. There was a difference in SiC for 1–3 and for 4‐ to 5‐year‐olds, which was higher but did not differ statistically 2009–2019. Among 1‐ to 3‐year‐olds, 32.1% were assessed as caries‐free. Among 4‐ to 5‐year‐olds, 16.0% were caries‐free. There was a statistically significant, lower number of caries‐free 1‐ to 3‐year‐old and 4‐ 5‐year‐old children 2009–2019.

**Conclusions:**

The mean dmft has been found to be continuously high from 2009 to 2019. The SiC score remained unchanged, whereas pufa was significantly lower. The frequency of caries‐free children was lower over time.

## Graphical abstract

Over a 10‐year period (2009–2019), 5090 children (1–5 years) in preschools in informal settlements outside Cape Town were examined for Early Childhood Caries. The findings highlight a significant need for dental care: The mean dmft was high, 4.33 for 1‐ to 3‐year‐olds and 6.34 for 4‐ to 5‐year‐olds with an increase over time. SiC scores remained high. Although pufa decreased, only 32.1% of younger and 16.0% of older children were caries‐free. The proportion of caries‐free children dropped notably over the decade.

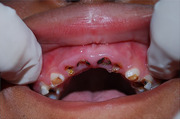

## 1 Introduction

Dental caries is the most common non‐communicable disease, affecting up to 90% of schoolchildren worldwide. As a result, the WHO recognizes it as a significant global burden [1, 2]. Research indicates that, regardless of a country's economic resources or healthcare infrastructure, dental caries leads to extensive human suffering that must be addressed [3]. For caries in primary teeth in children under 6 years of age, known as Early Childhood Caries (ECC), has been defined in various ways over the years. However, in 2019, during a meeting of the International Association for Pediatric Dentistry, ECC was unanimously defined as *“The presence of one or more decayed surfaces in any primary tooth of a child under six years of age*.*”* Consequently, the previous classification of Severe Early Childhood Caries (S‐ECC), which applied to children aged 3–5, was abolished. This change underscores the serious risk of caries in preschool‐aged children [4].

Decades of research indicate that ECC affects children in both welfare states and developing countries, though its distribution is highly uneven across populations [5, 6]. In a study of preschool children in Johannesburg, South Africa, Mothupi et al. reported a mean dmft score of 2.4 ± 3.4, with a Significant Caries Index (SiC) score exceeding 5 [7]. The substantial gap between the mean dmft and SiC scores suggests significant skewness in caries distribution. A systematic review by Kimmie‐Dhansay et al. confirmed this trend across 21 eligible studies [8]. Children living in informal settlements are particularly vulnerable to ECC, often experiencing severe suffering as a result [7, 8, 9]. For comparison, data from Sweden shows an incidence of caries, expressed as defs >0, in the age group 1–5 years to vary between 1% for the 1‐year‐olds and 23% for the 5‐year‐olds [3]. The youngest children are the most severely impacted, particularly in terms of physical growth and learning abilities [10, 11]. Ultimately, ECC affects children in both welfare states and developing countries, contributing to significant human suffering that must be addressed [3].

Extensive research on ECC has identified several key risk factors, including frequent sugar consumption, infrequent toothbrushing, low fluoride exposure, maternal education level, and genetic predisposition [12, 13, 14]. A low level of parental education has been strongly correlated with a higher prevalence of ECC in young children. Moreover, socioeconomic status has been shown to play a crucial role in enabling, initiating, and sustaining ECC in millions of young children [3, 15]. Given its multifactorial nature, ECC is likely influenced by socioeconomic conditions, overall health status, and access to oral healthcare, particularly among South Africa's most vulnerable children [16]. Additionally, the widespread occurrence of ECC places a significant strain on national healthcare systems [8].

The dmft index is the most commonly used measure for assessing caries in primary teeth [17]. However, because caries distribution is often skewed within populations, the SiC index was developed to provide a more accurate representation. This index calculates the mean dmft value for the one‐third of the population with the highest caries experience [18]. To further assess the impact of untreated dental caries in young children, the pufa index is used. This index identifies visible signs of severe dental infection, including pulp involvement (p), ulceration (u), fistula (f), and abscess (a) [19, 20]. By utilizing these standardized indices, countries can assess and track the oral health status of children over time. According to the WHO, dental examinations conducted under field conditions provide an adequate assessment of caries prevalence [21].

Because the most vulnerable children in informal settlements often experience a high burden of ECC, the impact falls not only on the individual but also on the healthcare system. Given the available data on oral health among vulnerable children in South Africa, there has been a longstanding need to study ECC prevalence using dmft, pufa, and SiC indices over an extended period [5, 13, 22]. This would enable relevant authorities to implement targeted measures to promote oral health.

## 2 Aim

This series of studies aimed to assess the prevalence of ECC by measuring dmft, SiC, pufa, and caries‐free status in informal settlements outside Cape Town, South Africa, over a 10‐year period.

## 3 Materials and Methods

### 3.1 Study Set‐Up

A series of cross‐sectional studies by student research teams over a 10‐year period (2009–2019) recorded ECC assessments in separate independent survey. A research collaboration between the University of Gothenburg, Sweden, and the University of the Western Cape, South Africa, surveys were carried out in informal settlements southeast of Cape Town (Table 1). In each settlement, three to seven preschools were visited. During the 10‐year period, 5090 children, 1–5 years of age were examined.

**TABLE 1:** Clinical caries assessments were performed in five informal settlements outside Cape Town.

Years	Settlement	1–3 years	4–5 years	1–5 years
		*n*	*n*	*n* total
2009	Khayelitsha	84	217	301
2010	Khayelitsha	145	148	293
2011	Khayelitsha	89	64	153
2012	Khayelitsha	156	197	353
2012	Broadlands Park	40	204	244
2012	Broadlands Park	92	159	251
2013	Khayelitsha	88	106	194
2013	Broadlands Park	78	219	297
2013	Broadlands Park	40	217	257
2014	Broadlands Park	84	207	291
2014	Broadlands Park	85	206	291
2014	Broadlands Park	50	138	188
2015	Broadlands Park	43	139	182
2016	Broadlands Park	38	134	172
2016	Gugulethu	36	61	97
2017	Nomzamo	103	193	296
2017	Broadlands Park	13	205	218
2018	Nomzamo	72	235	307
2018	Broadlands Park	9	151	160
2019	Nomzamo	118	84	202
2019	Broadlands Park	6	122	128
2019	Mitchell´s Plain	38	177	215
	**Total**	**1507**	**3583**	**5090**

*Note:* In total, 5090 children were orally screened for caries and pufa in their primary teeth through the years 2009–2019 in 5 informal settlements during 22 visits outside Cape Town, South Africa. A total of 1507 of the children were 1–3 years old, whereas 3583 were between 4 and 5 years old.

### 3.2 Student Researchers

The research was carried out by dental students and dental hygienist students completing their master and bachelor theses, respectively, at the Institute of Odontology, Sahlgrenska Academy, University of Gothenburg, Sweden. In total, 22 teams, 1–3 student teams per year, took part in this exchange program with the University of the Western Cape, South Africa.

### 3.3 Calibration to Measure dmft and pufa Prior to Data Sampling

All students took part in caries calibrations, consisting of 10 clinical image cases. The images (from preschool children in Khayelitsha and Broadlands Park) displayed visible primary dentition caries of varying degrees, in addition to pulp involvement, fistulas, and abscesses with dental origin. The calibration exercises continued on a weekly basis until the inter‐examiner reliability and the agreement versus the “gold standard” (the author TL) exceeded 90%.

### 3.4 Clinical Caries Assessment

Students followed a dental hygienist schedule in the informal settlements (Table 1). The children that could demonstrate informed consent documents were included in the study. Clinical caries assessments were performed according to a simplified WHO survey form for field studies [21]. Indoor illumination and sunlight were the light sources, and no radiographs were used. The intraoral examinations were carried out using examination mirrors. In some cases, clinical photos documented the oral status.

Each primary tooth was diagnosed with 1 dmft variable, allowing a maximum dmft score per mouth of 20. Primary teeth that had been extracted were recorded as missing due to caries. Preschool children with a zero dmft score were recorded as caries‐free, even without x‐rays [17]. SiC was calculated using one third of the highest dmft scores [18].

For the pufa index, pulp involvement (p) was recorded when a pulp chamber was exposed or if only root residues remained. Ulceration (u) was recorded when a carious dislocated tooth fragment caused an ulceration to the mucosa. Fistula (f) was recorded when a visible drain caused by an infected tooth root was found. Abscess (a) was registered when a swelling of pus associated with an infected tooth root was present. Each primary tooth was attributed one pufa variable, regardless of the number of variables present [19].

### 3.5 Ethical Considerations

The project was approved by the University of Western Cape, Biomedical Research Ethics Committee. Prior to the study start, project information and consent forms were given to the concerned legal guardians in English and the local Xhosa language. To be enrolled in the study, each child needed to give their verbal permission, in addition to the parent or guardian's consent.

The participants’ name, age, and gender were collected, but anonymity was ensured for all subsequent data processing. Participation was voluntary, so the children and their legal guardians could withdraw their participation at any time without giving a reason.

### 3.6 Statistics

The 10‐year data for the 1‐ to 3‐year‐old group and the 4‐ to 5‐year‐old group were analyzed separately. Mean scores were calculated for the dmft, SiC, and pufa variables, respectively. Regression lines, goodness of fit, and statistical differences from the horizontal zero slope were computed using the GraphPad statistical package. A *p* value < 0.05 was considered to be a statistically significant difference.

## 4 Results

In total, 5090 children were orally screened for caries and pufa in their primary teeth, through the years 2009–2019 during 22 informal settlement visits outside Cape Town, South Africa. 1507 (29.6%) of the children were 1–3 years old, whereas 3583 (70.4%) were between 4 and 5 years old (Table 1).

### 4.1 dmft

More than 50% of all the examined children had one or more decayed upper central primary incisors. The mean dmft was 5.5 for boys and 5.1 for girls.

**FIGURE 1:**
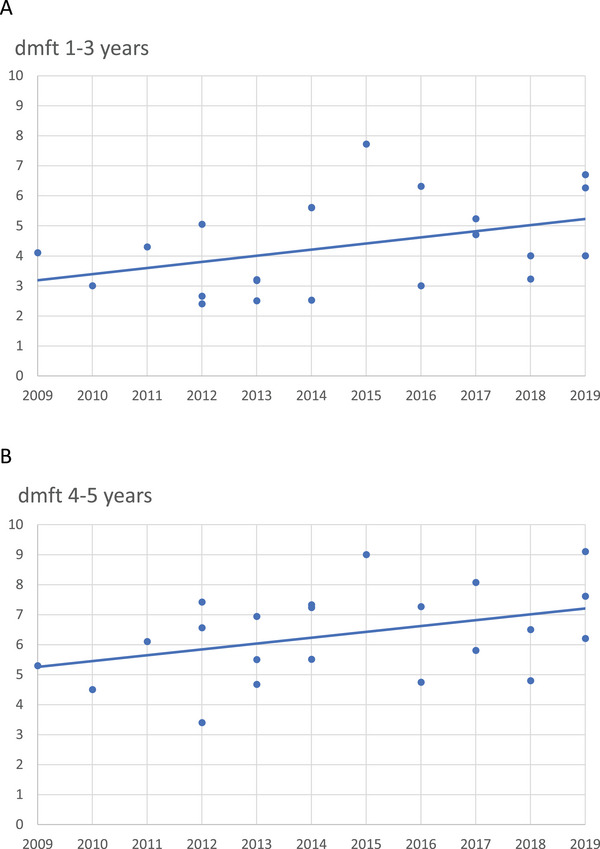
(A) The difference in dmft for 1‐ to 3‐year‐olds was lower but did not differ statistically between 2009 and 2019. The line of regression did not differ statistically (*p* > 0.05) from the horizontal zero slope. One dot represents the dmft average from one set of recordings in one settlement. (B) There was a significantly lower dmft for 4‐ and 5‐year‐olds between 2009 and 2019. The line of regression differed statistically (*p* < 0.05) from the horizontal zero slope. One dot represents the dmft average from one set of recordings in one settlement.

For 1‐ to 3‐year‐olds, the mean dmft was 4.33 ± 1.55, and the median was 4.05. For this group, the different dmft's over time did not vary statistically (*p* < 0.05) (Figure 1A).

For 4‐ and 5‐year‐olds, the mean dmft was 6.34 ± 1.48, and the median was 6.35. For them, the line of regression differed statistically from the horizontal zero slope, showing a significantly higher dmft over time (*p* < 0.05) (Figure 1B).

### 4.2 pufa

**FIGURE 2:**
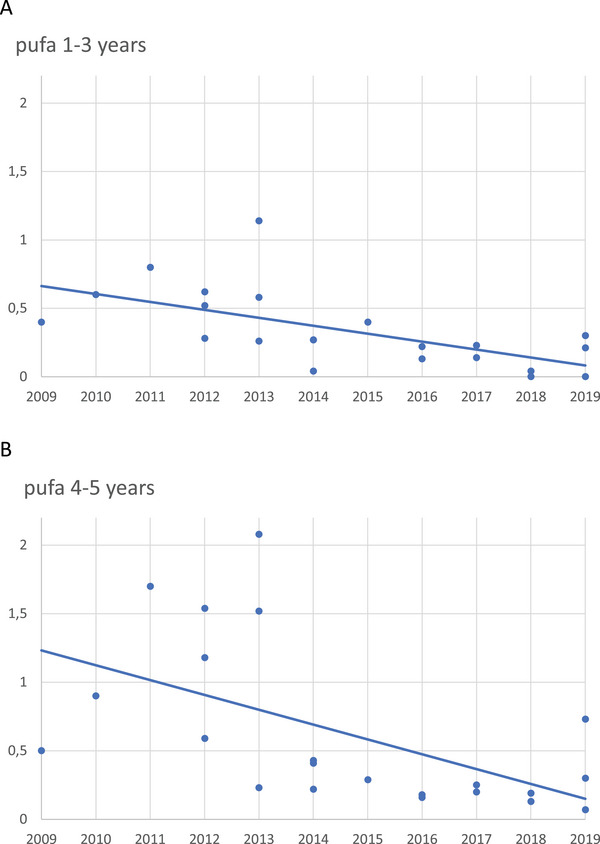
(A) For 1‐ to 3‐year‐olds, there was a significantly lower pufa between 2009 and 2019. The line of regression differed statistically (*p* < 0.05) from the horizontal zero slope. One dot represents the pufa average from one set of recordings in one settlement. (B) For 4‐ and 5‐year‐olds, there was a significantly lower pufa between 2009 and 2019. The line of regression differed statistically (*p* < 0.05) from the horizontal zero slope. One dot represents the pufa average from one set of recordings in one settlement.

For 1‐ to 3‐year‐olds, the mean pufa was 0.34 ± 0.28, and the median was 0.27. Their line of regression showed a significantly lower pufa over time (*p* < 0.05) (Figure 2A).

For 4‐ to 5‐year‐olds, the mean pufa was 0.63 ± 0.59, and the median was 0.36. Their line of regression showed a significantly lower pufa over time (*p* < 0.05) (Figure 2B).

### 4.3 SiC

**FIGURE 3:**
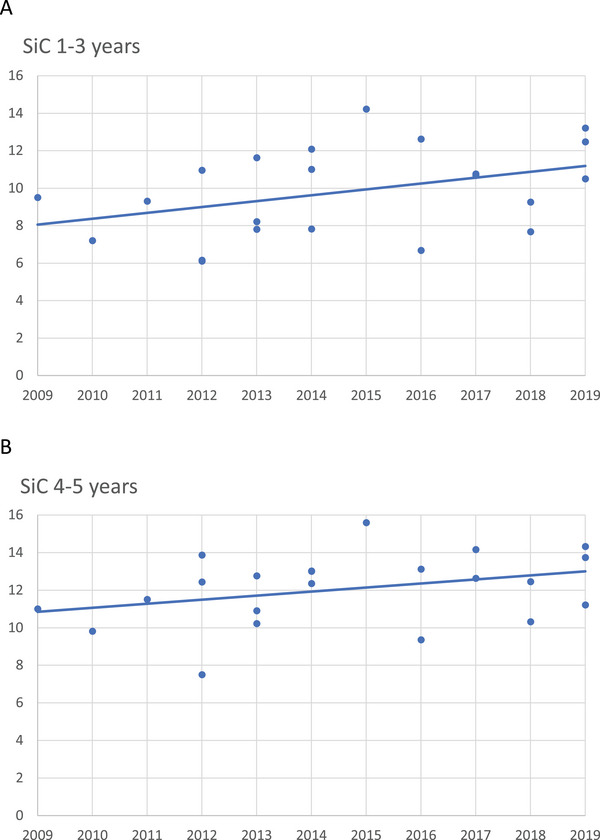
(A) The difference in SiC for 1‐ to 3‐year‐olds was higher but did not differ statistically between 2009 and 2019. The line of regression did not differ statistically (*p* > 0.05) from the horizontal zero slope. One dot represents the SiC average from one set of recordings in one settlement. (B) The difference in SiC for 4‐ and 5‐year‐olds was higher but did not differ statistically between 2009 and 2019. The line of regression did not differ statistically (*p* > 0.05) from the horizontal zero slope. One dot represents the SiC average from one set of recordings in one settlement. SiC, Significant Caries Index.

For 1‐ to 3‐year‐olds, the mean SiC was 11.0 ± 2.35, and the median was 11.0. Their different dmft's over time did not differ statistically (*p* > 0.05. NS) (Figure 3A).

For 4‐ to 5‐year‐olds, the mean SiC was 12.05 ± 1.89, and the median was 12.44. Their different dmft's over time did not differ statistically (>0.05. NS) (Figure 3B).

### 4.4 Caries‐Free

**FIGURE 4:**
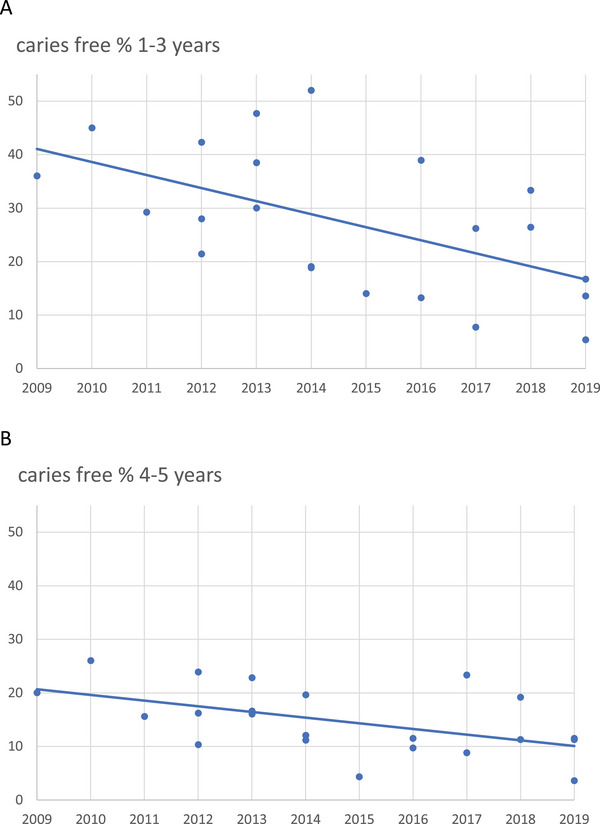
(A) There was a significantly lower number of caries‐free 1‐ to 3‐year‐old children between 2009 and 2019. The line of regression differed statistically (*p* < 0.05) from the horizontal zero slope. One dot represents the dmft average from one set of recordings in one settlement. (B) There was a significantly lower number of caries‐free 4 and 5‐year‐old children between 2009 and 2019. The line of regression differed statistically (*p* < 0.05) from the horizontal zero slope. One dot represents the dmft average from one set of recordings in one settlement.

Among 1‐ to 3‐year‐olds, 32.1% were assessed being caries‐free, and there was a significantly lower number of caries‐free children over time (*p* < 0.05) (Figure 4A).

Among 4‐ to 5‐year‐olds, 16.0% were assessed as being caries‐free, and there was a significantly lower number of caries‐free children over time (*p* < 0.05) (Figure 4B).

## 5 Discussion

In order to understand a major oral health problem and plan for improvement measures, a 10‐year‐long repeated cross‐sectional study was performed. The main results from this study demonstrate that ECC constitutes a substantial problem for children living in the informal settlements near Cape Town, South Africa. The findings furthermore indicate that ECC has been found to be continuously high between 2009 and 2019. The strength of this project is its unique duration and repeated study format, that is, a 10‐year‐long repeated cross‐sectional study, which enables time‐related ECC assessments and differences over a long period. An additional strength is the large sample size of preschool children that were scanned for ECC. More than 5000 children were orally screened for caries and pufa in their primary teeth during the 10‐year period. To the authors’ knowledge, there is no 10‐year prospective study in which ECC is followed and evaluated to this extent.

Every year, the Western Cape Provincial Parliament publishes Annual Performance Plans outlining the goals and strategies for the upcoming year. Children and child healthcare are recurring topics in these plans. Caries and measures to combat caries in children were last mentioned in the 2011–2012 Annual Performance Plan. Although a high prevalence of caries has been recognized among children living in informal settlements, there is a lack of current data and trends over time. The Western Cape province is home to over 6 million people, with an estimated 700,000 children aged 1–5 in 2019. The exact number of children living in informal settlements outside Cape Town remains unclear. Additionally, there are approximately 100 public health dentists and dental specialists in the province, creating a high ratio of children to available dentists. Overall, there is a clear need for up‐to‐date caries epidemiological data to inform healthcare planning [23].

Postma et al. [9] point out that the significant social divides in South Africa have led to a considerably higher incidence of caries in the primary dentition among Black and Colored children compared to White children. The authors also discuss the importance of reaching the most disadvantaged groups in South Africa through an integrated primary oral healthcare approach. They argue that this approach should not only include oral health promotion interventions but also address underlying social determinants [9]. In a review by Kimmie‐Dhansay et al., the researchers found that dmft values varied between 0.33 in Limpopo and 3.85 in the Eastern Cape, two similarly deprived regions of South Africa [8]. A parallel can be drawn to Sweden, where there are considerable differences in mean dmft values between socioeconomically advantaged and disadvantaged areas, particularly among the youngest preschool children. Anderson et al. [24] attempted to address this unjust and preventable burden by educating mothers of children under the age of three. However, despite biannual instructions and fluoride varnish treatments for the affected children, the educational efforts were unsuccessful. This outcome highlights the complexity of caries, which is influenced by factors such as cultural habits, family attitudes, and various immediate environmental influences. These factors may overshadow the effects of maternal education and socioeconomics, which are deeply rooted in culture and difficult to change [24].

In the present study, the mean dmft for 1‐ to 3‐year‐olds was 4.33 ± 1.55. Within this age group, dmft values did not show statistically significant variation over time. For 4‐ and 5‐year‐olds, the mean dmft was 6.34 ± 1.48. In this group, the regression line differed statistically from a horizontal zero slope, indicating a significant increase in dmft over time. Among all children aged 1–5 years, there was a significant decline in the number of caries‐free children over time, suggesting that the overall caries burden increased during the study period. These findings indicate that the caries situation among older preschool children is gradually worsening, despite the presence of a health program in which a dental hygienist regularly visits preschools to provide dietary information and apply fluoride varnish. The overall caries prevalence in the current study was 72.6%, which aligns with previous findings on schoolchildren in South Africa, where prevalence rates as high as 70% have been reported [10]. However, a national survey conducted in 2022 by Kimmie‐Dhansay et al. reported an overall dmft score of 2.42, which is lower than the findings of the present study [8].

For 1‐ to 3‐year‐olds, the mean SiC was 11.0 ± 2.35, whereas for 4‐ to 5‐year‐olds, it was 12.05 ± 1.89. The large difference between dmft and SiC suggests a skewed distribution of caries occurrence, where fewer children experience a disproportionately higher prevalence of caries. There was no statistically significant change in the mean SiC score for the most affected children over the study period. A survey by Postma et al., conducted in seven provinces of South Africa, reported a mean SiC score of 7.6 [9]. In contrast, the present study found a notably higher mean SiC value of 12.05.

For all children in the present study, the mean caries prevalence was 72.6%, reflecting the phenomenon of ECC. Despite a global decline in caries prevalence, the incidence of ECC among children living in poverty has continued to rise [25, 26]. Each country must address the determinants of ECC based on its own domestic circumstances, resources, and regulations. It can be assumed that, regardless of the structure of a country's dental care system, the most vulnerable children remain equally disadvantaged in terms of oral health. Although published studies report various methods and findings from diverse young South African populations, they consistently highlight the magnitude of the ECC problem. In the present study, the mean dmft, reflecting ECC among children in informal settlements in the Western Cape, remained persistently high throughout the 10‐year study period. Kazeminia et al. reported an overall global prevalence of ECC in primary teeth at 46.2% [27]. Similarly, Kimmie‐Dhansay et al. reported an ECC prevalence of 44.94% in South Africa, which is notably lower than the findings of the present study [8]. Postma et al. reported an ECC prevalence of all severity grades at 55%, also lower than in the current study [9]. The caries situation among children in Cape Town's informal settlements is alarming.

Untreated caries and its complications were measured using the pufa score. For 1‐ to 3‐year‐olds, the mean pufa was 0.34 ± 0.28, with the regression line showing a statistically significant decline over time. Similarly, for 4‐ to 5‐year‐olds, the mean pufa was 0.63 ± 0.59, and the regression analysis also indicated a statistically significant decrease over time for this group. Among all indicators, pufa most clearly reflected the extent of suffering. Although the mean dmft score increased with age, the pufa score simultaneously declined over time. This may seem contradictory but could indicate improved access to emergency dental care for affected children during the study period. The pufa score must always be assessed in the context of dental care availability, which is particularly relevant for South African children. To the authors’ knowledge, no other South African pufa values have been reported outside of the present study. As a comparison, the mean pufa value for Nigerian 4‐ to 6‐year‐olds was found to be 0.16, which is considerably lower than in the present study [28]. In contrast, Pakistani 4‐ to 5‐year‐olds had a mean pufa value of 0.86, which was higher than both the Nigerian value and the findings of the present study [29].

The caries situation in shantytowns not only affects children on an individual level but also places a significant burden on the healthcare system and society as a whole. Given the available data on oral health status among vulnerable child populations in South Africa, there has been a longstanding need to study ECC prevalence over an extended period using dmft, as well as pufa and SiC [5, 13, 22]. With this in mind, the relevant authorities could be given the opportunity to implement appropriate oral health promotion measures.

## 6 Conclusions

The mean dmft, reflecting ECC among children in South African informal settlements, remained consistently high from 2009 to 2019. Over time, caries prevalence increased among all children, whereas the mean pufa decreased, and the SiC score remained unchanged. The proportion of caries‐free children was low. The caries burden in this vulnerable population is significant and demands urgent attention to the known determinants of ECC. Addressing this issue requires well‐established methods of dental care planning, disease prevention, and containment.

## Author Contributions

**Larisa Krekmanova**: Data Curation, Validation, Visualization, Writing Original Draft Preparation. **Neil Myburgh**: Funding Acquisition, Investigation, Methodology, Project Administration, Resources, Supervision, Writing Review & Editing. **Ted Lundgren**: Conceptualization, Data Curation, Formal Analysis, Funding Acquisition, Investigation, Writing Original Draft Preparation.

## Ethics Statement

The authors declare that the investigation was carried out following the rules of the Declaration of Helsinki of 1975, revised in 2013. According to point 23 of this declaration, the study and the protocols used were approved by the University of the Western Cape, Biomedical Research Ethics Committee. Prior to the study start, project information and consent forms were given to the concerned legal guardians in English and the local Xhosa language. To be enrolled in the study, each child needed to give their verbal permission, in addition to the parent or guardian's consent.

The participants’ name, age, and gender were collected, but anonymity was ensured for all subsequent data processing. Participation was voluntary, so the children and their legal guardians could withdraw their participation at any time without giving a reason. A total of 5090 children aged from 1 to 5 years of age were examined between 2009 and 2019. The caries parameters dmft (decayed, missing, and filled teeth) and pufa (pulp exposure, ulcerations, fistulas, and abscesses) were assessed.

## Conflicts of Interest

The authors declare no conflicts of interest.

## Data Availability

The data that support the findings of this study are available on request from the corresponding author. The data are not publicly available due to privacy or ethical restrictions.

## References

[1] S. E. Uribe , N. Innes , and I. Maldupa , “The Global Prevalence of Early Childhood Caries: A Systematic Review With Metaanalysis Using the WHO Diagnostic Criteria,” International Journal of Paediatric Dentistry 31 (2021): 817–830. 33735529 10.1111/ipd.12783

[2] P. E. Petersen , D. Bourgeois , D. Bratthall , and H. Ogawa , “Oral Health Information Systems—Towards Measuring Progress in Oral Health Promotion and Disease Prevention,” Bulletin of the World Health Organization 83 (2005): 686–693. 16211160 PMC2626332

[3] M. Anderson , G. Dahllöf , A. Warnqvist , and M. Grindefjord , “Development of Dental Caries and Risk Factors Between 1 and 7 Years of Age in Areas of High Risk for Dental Caries in Stockholm, Sweden,” European Archives of Paediatric Dentistry 22 (2021): 947–957. 34106458 10.1007/s40368-021-00642-1 PMC8526475

[4] “Early Childhood Caries: IAPD Bangkok Declaration,” International Journal of Paediatric Dentistry 29 (2019): 384–386. 31099129 10.1111/ipd.12490

[5] S. Weston‐Price , V. Copley , H. Smith , and G. M. Davies , “A Multi‐Variable Analysis of Four Factors Affecting Caries Levels Among Five‐Year‐Old Children; Deprivation, Ethnicity, Exposure to Fluoridated Water and Geographic Region,” Community Dental Health 29 (2018): 217–222. 10.1922/CDH_4383Weston-Price06 30188616

[6] M. D. Macek , K. H. Heller , R. H. Selwltz , and M. C. Manz , “Is 75 Percent of Dental Caries Really Found in 25 Percent of the Population?” Journal of Public Health Dentistry 64 (2004): 20–25. 15078057 10.1111/j.1752-7325.2004.tb02721.x

[7] K. A. Mothupi , C. B. Nqcobo , and V. Yengopal , “Prevalence of Early Childhood Caries Among Preschool Children in Johannesburg, South Africa,” Journal of Dentistry for Children (Chicago, Ill.) 83 (2016): 83–87. 27620519

[8] F. Kimmie‐Dhansay , R. Barrie , S. Naidoo , and T. Roberts , “Prevalence of Early Childhood Caries in South Africa: A Systematic Review,” BMC Oral Health 22, no. 1 (2022): 32. 35135513 10.1186/s12903-021-01982-6 PMC10074718

[9] C. T. Postma O. A. Ayo‐Yusuf , and P. J. van Wyk , “Socio‐Demographic Correlate: Of Early Childhood Caries Prevalence and Severity in a Developing Country‐ South Africa,” International Dental Journal 58 (2008): 91–97. 18478890 10.1111/j.1875-595x.2008.tb00182.x

[10] S. Naidoo and N. Myburgh , “Nutrition, Oral Health and the Young Child,” Maternal & Child Nutrition 3 (2007): 312–321. 17824859 10.1111/j.1740-8709.2007.00115.x PMC6860753

[11] A. Saikia , J. Aarthi , M. S. Muthu , et al., “Sustainable Development Goals and Ending ECC as a Public Health Crisis,” Frontiers in Public Healthh 18 (2022): 931243. 10.3389/fpubh.2022.931243 PMC9624450 36330110

[12] W. Kim Seow , “Environmental, Maternal, and Child Factors Which Contribute to Early Childhood Caries: A Unifying Conceptual Model,” International Journal of Paediatric Dentistry 22 (2012): 157–168. 21972925 10.1111/j.1365-263X.2011.01186.x

[13] L. G. Do , J. A. Scott , W. M. Thomson , et al., “Common Risk Factor Approach to Address Socioeconomic Inequality in the Oral Health of Preschool Children—A Prospective Cohort Study,” BMC Public Health [Electronic Resource] 6 (2014): 429. 10.1186/1471-2458-14-429 PMC4039048 24885129

[14] A. M. Nunes , C. M. Alves , F. Borba de Araújo , et al., “Association Between Prolonged Breast‐Feeding and Early Childhood Caries: A Hierarchical Approach,” Community Dentistry and Oral Epidemiology 40 (2012): 542–549. 22725605 10.1111/j.1600-0528.2012.00703.x

[15] J. A. Weatherwax , K. K. Bray , K. B. Williams , and C. C. Gadbury‐Amyot , “Exploration of the Relationship Between Parent/Guardian Sociodemographics, Intention, and Knowledge and the Oral Health Status of Their Children/Wards Enrolled in a Central Florida Head Start Program,” International Journal of Dental Hygiene 13 (2015): 49–55. 25040842 10.1111/idh.12097

[16] A. Simón‐Soro and A. Mira , “Solving the Etiology of Dental Caries,” Trends in Microbiology 23 (2015): 76–82. 25435135 10.1016/j.tim.2014.10.010

[17] J. M. Broadbent and W. M. Thomson , “For Debate: Problems With the DMF Index Pertinent to Dental Caries Data Analysis,” Community Dentistry and Oral Epidemiology 33 (2005): 400–409. 16262607 10.1111/j.1600-0528.2005.00259.x PMC1388190

[18] D. Bratthall , “Introducing the Significant Caries Index Together With a Proposal for a New Global Oral Health Goal for 12‐Year‐Olds,” International Dental Journal 50 (2000): 378–384. 11197197 10.1111/j.1875-595x.2000.tb00572.x

[19] B. Monse , R. Heinrich‐Weltzien , H. Benzian , C. Holmgren , and W. van Palenstein Helderman , “PUFA—An Index of Clinical Consequences of Untreated Dental Caries,” Community Dentistry and Oral Epidemiology 38 (2010): 77–82. 20002630 10.1111/j.1600-0528.2009.00514.x

[20] A. B. S. Lopes , M. L. Ramos‐Jorge , G. F. Machado , R. G. Vieira‐Andrade , J. Ramos‐Jorge , and I. B. Fernandes , “Longitudinal Evaluation of Determinants of the Clinical Consequences of Untreated Dental Caries in Early Childhood,” Community Dentistry and Oral Epidemiology 50 (2022): 91–98. 33704820 10.1111/cdoe.12635

[21] P. E. Petersen and R. J. Baez , Oral Health Surveys: Basic Methods, 5th ed. (World Health Organization, 2013).

[22] R. Harris , A. D. Nicoll , P. M. Adair , and C. M. Pine , “Risk Factors for Dental Caries in Young Children: A Systematic Review of the Literature,” Community Dental Health 21, no. S1 (2004): 71–85. 15072476

[23] Annual Performance Plan 2024–2025 . Western Cape Government. Department of Health and Wellness, https://www.westerncape.gov.za/health‐wellness/files/wcg‐blob‐files?file=2024‐10/wcghw_annual_performance_plan_app_2024‐2025.pdf&type=file.

[24] M. Anderson , “Stop Caries Stockholm: A Caries‐Prevention Program for Children Living in Multicultural Areas With Low Socioeconomic Status,” (Thesis, Department of Dental Medicine, Karolinska Institutet, 2017).

[25] W. J. Psoter , D. G. Pendrys , D. E. Morse , H. Zhang , and S. T. Mayne , “Associations of Ethnicity/Race and Socioeconomic Status With Early Childhood Caries Patterns,” Journal of Public Health Dentistry 66 (2006): 23–29. 16570747 10.1111/j.1752-7325.2006.tb02547.x

[26] C. M. Vargas and C. R. Ronzio , “Disparities in Early Childhood Caries,” BMC Oral Health 15, no. S1 (2006): S3. 10.1186/1472-6831-6-S1-S3 PMC2147596 16934120

[27] M. Kazeminia , A. Abdi , S. Shohaimi , et al., “Dental Caries in Primary and Permanent Teeth in Children's Worldwide, 1995 to 2019: A Systematic Review and Meta‐Analysis,” Head & Face Medicine 16 (2020): 22. 33023617 10.1186/s13005-020-00237-z PMC7541284

[28] E. O. Oziegbe and T. A. Esan , “Prevalence and Clinical Consequences of Untreated Dental Caries Using PUFA Index in Suburban Nigerian School Children,” European Archives of Paediatric Dentistry 14 (2013): 227–231. 23780656 10.1007/s40368-013-0052-5

[29] R. Kamran , W. Farooq , M. R. Faisal , and F. Jahangir , “Clinical Consequences of Untreated Dental Caries Assessed Using PUFA Index and Its Covariates in Children Residing in Orphanages of Pakistan,” BMC Oral Health 17 (2017): 108. 28693477 10.1186/s12903-017-0399-9 PMC5504620

